# Sex difference contributes to phenotypic diversity in individuals with neurodevelopmental disorders

**DOI:** 10.3389/fped.2023.1172154

**Published:** 2023-08-07

**Authors:** Tania Cuppens, Julie Shatto, Loïc Mangnier, Ajay A. Kumar, Andy Cheuk-Him Ng, Manpreet Kaur, Truong An Bui, Mickael Leclercq, Arnaud Droit, Ian Dunham, Francois V. Bolduc

**Affiliations:** ^1^ Centre de Recherche du CHU de Québec-Université Laval, Département de Médecine Moléculaire de L'Université Laval, Québec, QC, Canada; ^2^ Department of Pediatric Neurology, University of Alberta, Edmonton, AB, Canada; ^3^ European Molecular Biology Laboratory, European Bioinformatics Institute (EMBL-EBI); Wellcome Genome Campus, Cambridgeshire, United Kingdom; ^4^ Department of Medical Genetics, University of Alberta, Edmonton, AB, Canada; ^5^ Neuroscience and Mental Health Institute, University of Alberta, Edmonton, AB, Canada

**Keywords:** neurodevelopmental disorders, global developmental delay, sex differences, phenotype, genotype, autism spectrum disorder

## Abstract

**Objective:**

Gain a better understanding of sex-specific differences in individuals with global developmental delay (GDD), with a focus on phenotypes and genotypes.

**Methods:**

Using the Deciphering Developmental Disorders (DDD) dataset, we extracted phenotypic information from 6,588 individuals with GDD and then identified statistically significant variations in phenotypes and genotypes based on sex. We compared genes with pathogenic variants between sex and then performed gene network and molecular function enrichment analysis and gene expression profiling between sex. Finally, we contrasted individuals with autism as an associated condition.

**Results:**

We identified significantly differentially expressed phenotypes in males vs. females individuals with GDD. Autism and macrocephaly were significantly more common in males whereas microcephaly and stereotypies were more common in females. Importantly, 66% of GDD genes with pathogenic variants overlapped between both sexes. In the cohort, males presented with only slightly increased X-linked genes (9% vs. 8%, respectively). Individuals from both sexes harbored a similar number of pathogenic variants overall (3) but females presented with a significantly higher load for GDD genes with high intolerance to loss of function. Sex difference in gene expression correlated with genes identified in a sex specific manner. While we identified sex-specific GDD gene mutations, their pathways overlapped. Interestingly, individuals with GDD but also co-morbid autism phenotypes, we observed distinct mutation load, pathways and phenotypic presentation.

**Conclusion:**

Our study shows for the first time that males and females with GDD present with significantly different phenotypes. Moreover, while most GDD genes overlapped, some genes were found uniquely in each sex. Surprisingly they shared similar molecular functions. Sorting genes by predicted tolerance to loss of function (pLI) led to identifying an increased mutation load in females with GDD, suggesting potentially a tolerance to GDD genes of higher pLI compared to overall GDD genes. Finally, we show that considering associated conditions (for instance autism) may influence the genomic underpinning found in individuals with GDD and highlight the importance of comprehensive phenotyping.

## 1. Introduction

Sex differences in the prevalence of autism spectrum disorder (ASD)(3–4:1) (1) but also its phenotypic presentation have been described before (2–4) (“female protective”), underlining the importance of sex in clinical presentation of ASD (5). Nonetheless, there is a gap in our understanding of the role of biological, diagnostic, psychological, and social factors in this female camouflage (5–8). On the other hand, sex differences in other types of neurodevelopmental disorders (NDDs) have not been as extensively studied. Among NDDs, global developmental delay (GDD) is common, affecting up to 3% of the pediatric population (9, 10). GDD is diagnosed when an individual under the age of 5 fails to meet expected developmental milestones in 2 or more domains of development (11). Although diagnostic criteria for GDD are well-defined, variation in overall phenotypes, as well as presentation differences based on sex, have not been studied. Previous studies have shown a bias toward male individuals with GDD (2:1) (12), but did not investigate phenotypic differences This male bias also depended on the genes investigated (13, 14).

Here we leveraged the largest cohort of individuals with GDD, the Deciphering Developmental Disorders (DDD) cohort (15) in order to gain a deeper understanding of phenotypic diversity modified by sex in GDD. Importantly, the DDD cohort recruited individuals with unknown etiology after targeted assessment for more common disorders such as Fragile X syndrome and Angelman syndrome, and therefore represented an important clinical population. A complete portrait of sex differences in GDD should therefore also incorporate phenotypes from more common syndromes.

## 2. Materials and methods

### 2.1. Participants

We used the DDD data acquired from 24 clinical genetics centers within the United Kingdom (UK) National Health Service and the Republic of Ireland. Together, they recruited 13,462 individuals based on undiagnosed developmental disorders and their parents (9,860 trios) from April 2011 through April 2015 (16, 17). The inclusion criteria for the DDD study to recruit children is based on the evidence of neurodevelopmental disorder, congenital anomalies, abnormal growth parameters (height, weight, occipitofrontal circumference), dysmorphic features, unusual behavioral phenotype or genetic disorder of significant impact for which the molecular basis is currently unknown (18). After obtaining ethics approval at our centers, and with permission from the DDD consortium, we analyzed the dataset for phenotypes of the individuals. We downloaded and worked on the GrCh37 genome-aligned files from the last datafreeze of 2017-12-15.

The phenotypes listed in DDD follow the Human Phenotype Ontology (HPO) organization. The hierarchical structure of HPO can be accessed in two formats: OBO flat file format and Web Ontology Language (OWL). The OWL version is more enriched as it provides logical definitions of the HPO classes, which facilitates cross-species mapping with semantic reasoning tools (19). OWL format was used as it consists of existential and universal restrictions on HPO phenotype classes. Protégé, which is an open source ontology editor and framework (20), was used to explore the defined restrictions on different phenotypes. These restrictions describe anonymous ancestors of the phenotypes and were used to classify the phenotypes according to the body section. HPO presents the advantage of being a machine readable and cross-species usable ontology but has limitations when being used to characterize fine clinical phenotypes.

We identified individuals with phenotypes that included global developmental delay, which were then divided by sex. We excluded people whose sex or age was not specified, so a number of 6,988 individuals with GDD are retained for this study.

### 2.2. Genomic annotation

The exome sequence data of GDD phenotyped individuals were analyzed in two stages. In the first stage, the existing GRCh37/hg19 exome sequences were realigned to the GRCh38 genome reference sequence. Then the short variants (SNVs and indels) were called using GATK best practices workflow (21), which involves steps such as realigning reads to GRCh38 reference genome, variant calling using HaplotypeCaller and joint genotyping, and finally variant quality recalibration and refinement steps leading to a high quality variant callset. In the second stage, these variants were annotated for gene information (Ensembl), frequencies (from gnomAD, ExAC, and internal cohort GDD), and pathogenicity (from CADD, Clinvar, and Clingen). The annotated set of variants in the callset were filtered for gene information, rare variants having minor allele frequency (MAF) <=0.01, impact on the transcript, and pathogenicity (pathogenic/likely_pathogenic in CLINVAR/CLINSEG). The details of the annotation and filtering criteria can be found in the Supplementary data Section S1.1. For some analyses we considered genes with a higher probability of loss-of-function intolerance score (pLI >= 0.9).

### 2.3. Candidate gene list

In order to assess genomic data in a targeted manner, we developed disorder specific candidate gene lists. We searched PubMed using the keywords: intellectual disability (ID)” and “global developmental delay (GDD)”, reviewed the papers and compiled manually a list of genes from original research and review papers (22–25). We integrated both GDD and ID together as most papers reviewed did not make a clear distinction between the two. We also used genes included in already developed in trusted databases related to neurodevelopmental disorders (NDD) for diseases, phenotypes, and even genome wide association studies (GWAS) using the same keywords [SysID (26, 27), DisGenet (28, 29), HPO (19, 30), OMIM (31, 32), Orphanet (33), Phenolyzer (34, 35), Ingenuity Pathway Analysis (Qiagen), Open Targets (36, 37), AutDB (38)]. We also added the Intellectual Disability NGS Radboudumc and Fulgent gene panels to have the most complete overview. Each gene list was obtained separately, and then we retained only those genes that appeared at least 3 times in the collected data, resulting in a list of 2,539 candidate genes for ID/GDD (Supplementary Table S1a). We also similarly created a list of autism spectrum disorder (ASD) candidate genes. We searched PubMed using the keywords: “autism”, “autism spectrum disorder ASD”, and “autistic” and compiled a list of genes from original research and review papers (23, 39). To the database list used for ID + GDD gene list we added the database created by SFARI (40), resulting in a list of 730 genes (Supplementary Table S1b). We mainly based ourselves on curated databases from autism experts (SFARI) considering the issues with annotation which has been seen when using original reports (41). Several articles have already been screened by experts to be focused on ASD as some papers present genes related to autism but also associated with other traits. We are also using a stringent approach by using a threshold of 3 references per gene.

### 2.4. Expression data

Expression data was retrieved from GTEx v8 brain tissues to compare expression in males and females in brain regions (11 distinct brain regions and 2 cell lines) (42), using the medium Transcripts Per Million (TPM). We also used the BrainSpan data from both sexes, containing 19 brain regions (43). The median Reads Per Kilobase per Million (RPKM) was reported for each sex by tissue before and after birth.

### 2.5. Statistical analysis

We used logistic regression to highlight the differences between males and females with respect to phenotypes. Briefly, the logistic regression model estimates odds-ratios for binary outcomes. Only phenotypes with *N* > 5 in each category were retained in the analysis. Since we expected possible overlaps between phenotypes, due either to common causal mechanisms or phenotype misclassifications, we applied the Benjamini Yekutieli correction to adjust *p*-values with the false discovery rate (FDR) for the multiple comparisons. Adjusted *p*-values lower or equal to 0.05 were considered statistically significant. We next used a linear mixed model to take into account the age in the model for the adjustment of the difference of the phenotypes between the sexes.

Consistently, to highlight the differences between males and females regarding the mutations in candidate genes, we still used a logistic regression model. However, due to strong mutated gene imbalance between sexes, we observed a bias toward conservatism. To address this limitation, nominal *p*-values lower or equal to 0.05 were considered statistically significant.

To assess whether the gene expression is impacted either by sex or developmental stage, we took advantage of linear mixed model of the logarithm of gene expression conditioning on sex (reference = female) and sex specificity (reference = male specific) for GTEx data, and for sex (reference = female), sex specificity (reference = male specific) and developmental stage (reference = after-birth), when analyzing BrainSpan data. One advantage of linear mixed models over others approaches is to model the dependence structure of expression for the same gene, while removing unmeasured confounding factors. Thus, we considered a random intercept for each gene. It is worth noting that for these analyses we adjusted for multiplicity using Bonferroni correction. Adjusted *p*-values lower or equal to 0.05 were considered statistically significant.

Finally, cluster analysis was performed using hierarchical cluster analysis methods with Ward distance. We chose this metric since it minimizes the intra-cluster variance while maximizing the inter-cluster variance, giving clusters with genes of closed expression. Threshold was chosen in both males and females for each brain region, using a graphical criteria.

### 2.6. Network and pathway analysis

For each group (males or females with GDD or other phenotypes of interest) the genes harboring mutations were then analyzed by protein-protein interaction prediction, clustering, and pathway enrichment using STRING v11.5 with full STRING network, all active interaction sources and medium confidence (0.4) (44). The Markov Clustering Algorithm (MCL) was used to identify gene subnetworks (45). The inflation parameter of MCL was set to 1.5 (46). Functional enrichment analysis of each module was also performed in the STRING v11.5 database using the GO terms, REACTOME pathways, and KEGG pathways. The false discovery rate obtained from the functional enrichment analysis describes the degree of significance of the enrichment. The *p*-values were corrected for multiple testing in each category using the Benjamini-Hochberg procedure. Visualizations of protein-protein interactions and clusters were also obtained using STRING v11.5.

### 2.7. Gene-Ontology enrichment analyses

In order to capture biological pathways occurring in male specific and female specific gene clusters, we applied cluster-based gene-ontology enrichment analyses using the function compareCluster from clusterprofiler R package (47).

## 3. Results

It is important to consider that the Deciphering Developmental Disorder (DDD) database includes individuals with GDD who presented to the DDD research team after having had a standard clinical assessment where known genetic disorders or syndromes, for instance Fragile X syndrome (FXS) Angelman, or Rett syndrome (15). Nonetheless, undiagnosed individuals after targeted genetic analysis represent the majority of individuals with GDD (48), making this analysis valuable both clinically and neurobiologically. We began by mapping the number of individuals with GDD in the DDD cohort, along with their sex. Of the 13,462 individuals with developmental disabilities, we selected those for whom we had information on sex (13,407 in total). To make the selection on GDD we included the individuals who presented one of the following HPO terms: Global developmental delay (HP:0001263), Mild global developmental delay (HP:0011342), Moderate global developmental delay (HP:0011343) and Severe global developmental delay (HP:0011344). Individuals with GDD represent 49.13% of the DDD cohort (6588 probands). Among the GDD there were 3,837 males (58.3%) and 2,751 females (41.7%) suggesting an even distribution of GDD between males and females (in DDD 7,828 male (58.3%) and 5,611 female (41.7%)). The mean age of the probands was 7 years old (range, 0 to 82 years old) at the time of recruitment.

### 3.1. Phenotypic differences in GDD

**Figure 1:**
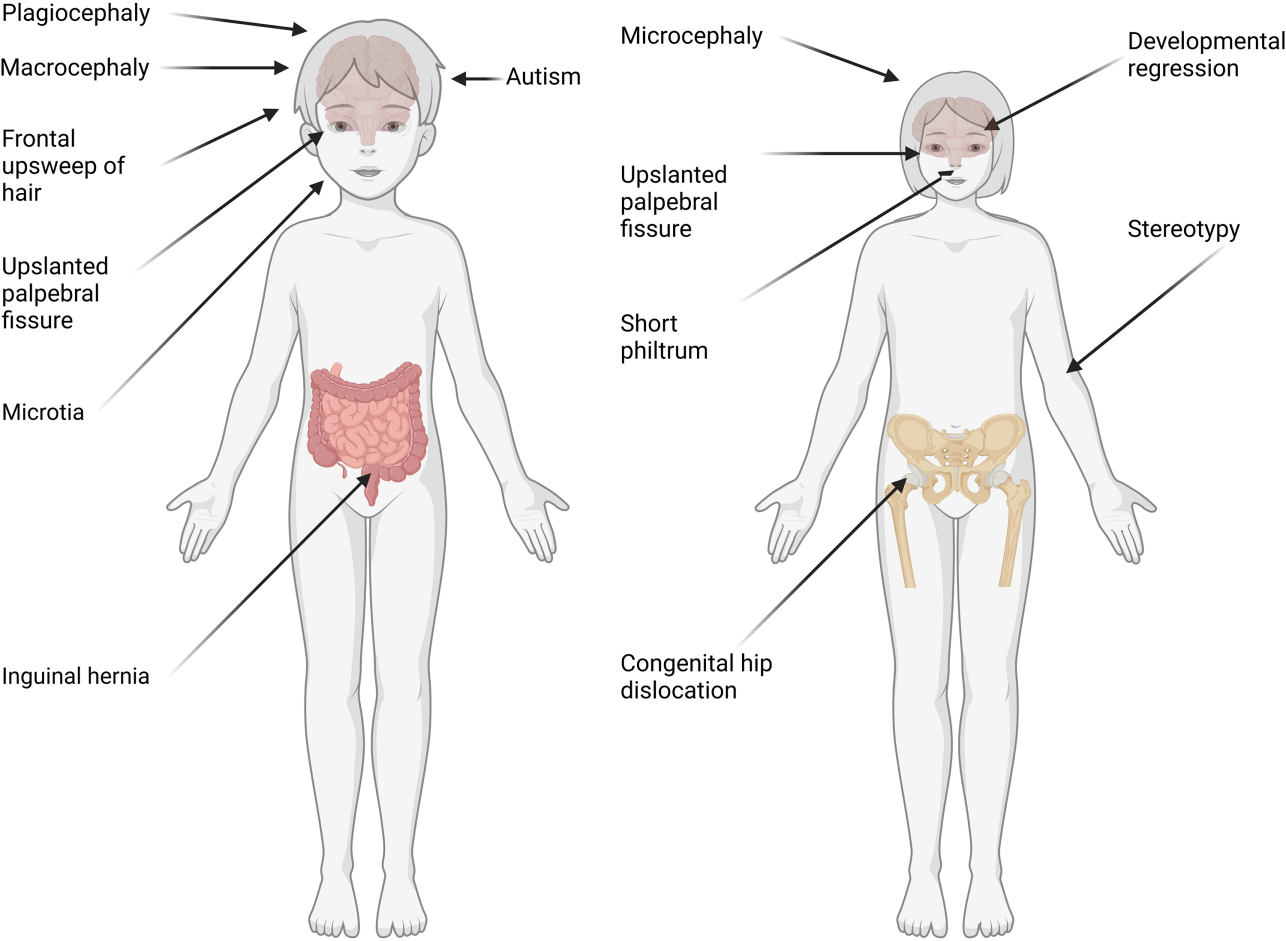
Representation of phenotypes based on sex in individuals with GDD. Phenotypes with significant differences (*p* value ≤ 0.05, *t*-test with Benjamini Yekutieli correction) between males (left) and females (right) are reported. Those include autism, macrocephaly and plagiocephaly in males. On the other hand, females presented with stereotypies, microcephaly and regression (probably related to inclusion of MECP2).

We observed significant differences between sex in several phenotypes in individuals with GDD (Figure 1, Supplementary Table S3). Indeed, we observed that males with GDD had a significantly higher rate of autistic behavior (HP:0000729, 7.57% males, 4.39% females, *P* = 9.82E-05) and autism (HP:0000717, 5.62% males, 3.09% females, *P* = 4.22E-04). We also found a significant increase in stereotypical movements (HP:0000733, 3.45% females, 1.54% males, *P* = 3.31E-04), short philtrum (HP:0000322, 2.87% females, 1.51% males, *P* = 4.70E-02), and congenital hip dislocation (HP:0001374, 0.94% females, 0.18% males, *P* = 7.15E-03) in females. There was a significant increase in frontal upsweep of hair (HP:0002236, 1.56% males, 0.40% females, *P* = 1.13E-03) and inguinal hernia (HP:0000023, 2.45% males, 0.25% females, *P* = 1.37E-11) in males. We also used logistic regression to assess whether there was a difference when adjusting for sex and age. We observed no significant differences after adjusting for multiple comparisons. Also, the *p* values of the interaction of the age through the sex were not significant. This means that there is insufficient evidence to conclude that the interaction of age through sex influences the phenotypes seen above (Supplementary Table S5).

Interestingly, we found several phenotypes where males and females presented with quantitatively or qualitatively “opposite” phenotypes (Figure 1). For example, down slanted palpebral fissure was increased in males (HP:0000494, 5.44% males, 3.23% females, *P* = 7.08E-03) whereas upslanted was increased in females (HP:0000582, 5.88% females, 3.25% males, *P* = 2.54E-04). Also, microcephaly was significantly more frequent in females (HP:0000252, 18.22% females, 12.88% males, *P* = 4.58E-06), while macrocephaly was significantly more common in males (HP:0000256, 4.29% males, 2.58% females, *P* = 4.70E-02). Developmental regression was significantly increased in females, probably due to the inclusion of individuals with Rett syndrome which is found most commonly in females (HP:0002376, 2.32% females, 1.07% males, *P* = 2.38E-022.38E-02).

### 3.2. Genotypic differences in GDD

**Figure 2:**
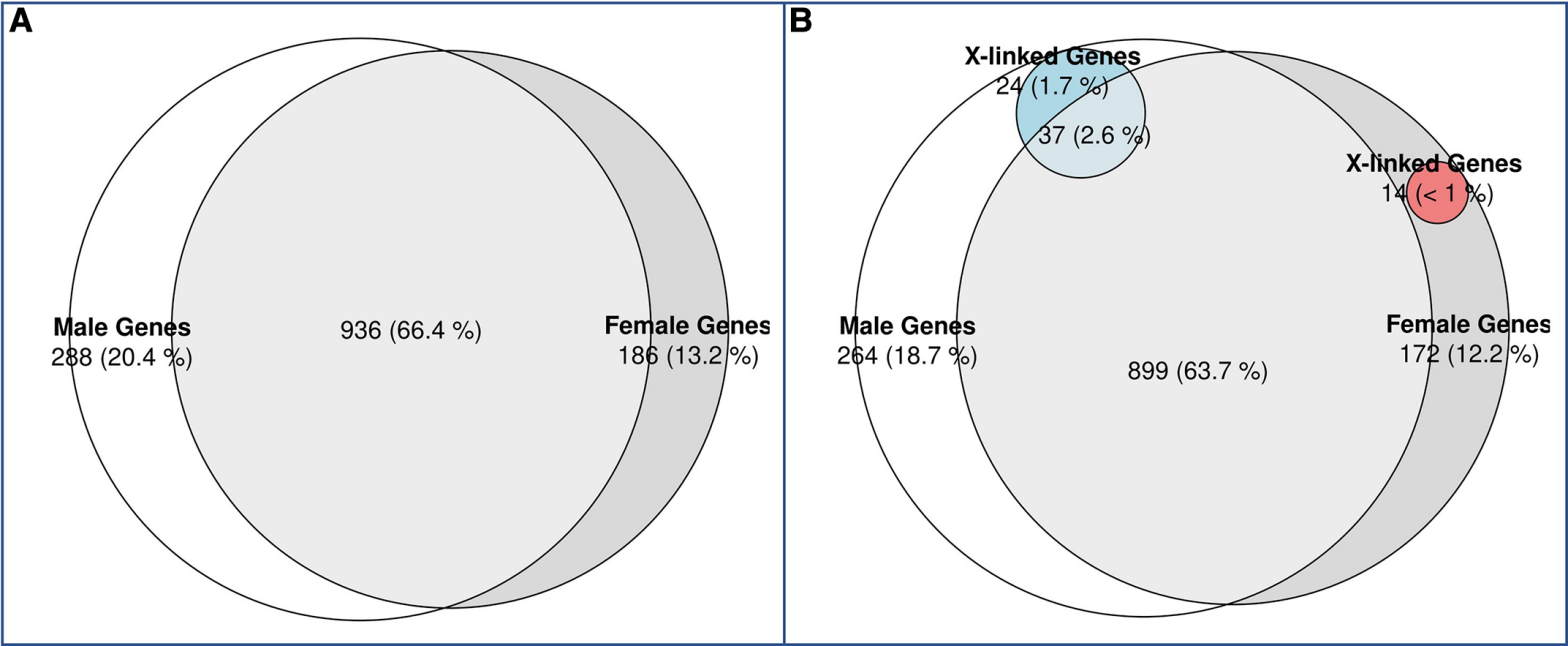
Venn diagram of candidate GDD genes identified in males vs. females with GDD. (**A**) While the majority of genes (66%) overlapped between males and females, 20% of GDD genes were found uniquely in males and 13% in females. (**B)** X-linked genes in males and females. X-linked genes accounted for 1.7% of male specific genes and 2.6% of shared genes between males and females. On the other hand, X-linked genes accounted for less than1% of female specific genes.

The rationale for DDD was to understand the genetic basis of undiagnosed individuals with NDD with more advanced genetic testing. Some individuals may have been included based on lack of clinical diagnosis (phenotypic presentation not fitting classical syndrome potentially) and then found to have mutations in genes syndromic. We did not exclude any condition, but the way in which the individuals in this database were recruited led us to the conclusion that there were no FXS in the cohort, as these were individuals with severe developmental delays who had not been diagnosed with any known disease. This could partly explain why we didn't observe a significant difference in the prevalence of GDD between the sexes, as *FMR1* is the most common single gene causing developmental differences, which is biased in favor of males. Indeed, we didn't identify any individuals carrying a pathogenic variant in the *FMR1* gene in the cohort. On the other hand, we found variants in the *MECP2* gene, which corresponds to Rett syndrome, although we believe that individuals with this syndrome were excluded from the initial cohort. We analyzed further differences in genetic mutations between sexes to gain a better understanding of differences in phenotypes. We identified likely pathogenic and pathogenic variants in the individuals with GDD using ClinVar (49). We found that 66.3% of GDD genes, affected by pathogenic or likely pathogenic variants, were found in both males and females (Figure 2A), but also identified genes (with pathogenic or likely pathogenic variants) specific to males (20.4%) and females (13.2%). We assessed if the pattern of male and female specific affected genes corresponded to X-linkage and found that 8.3% of male specific genes were X-linked compared to 7.5% in females (Figure 2B).

**Figure 3:**
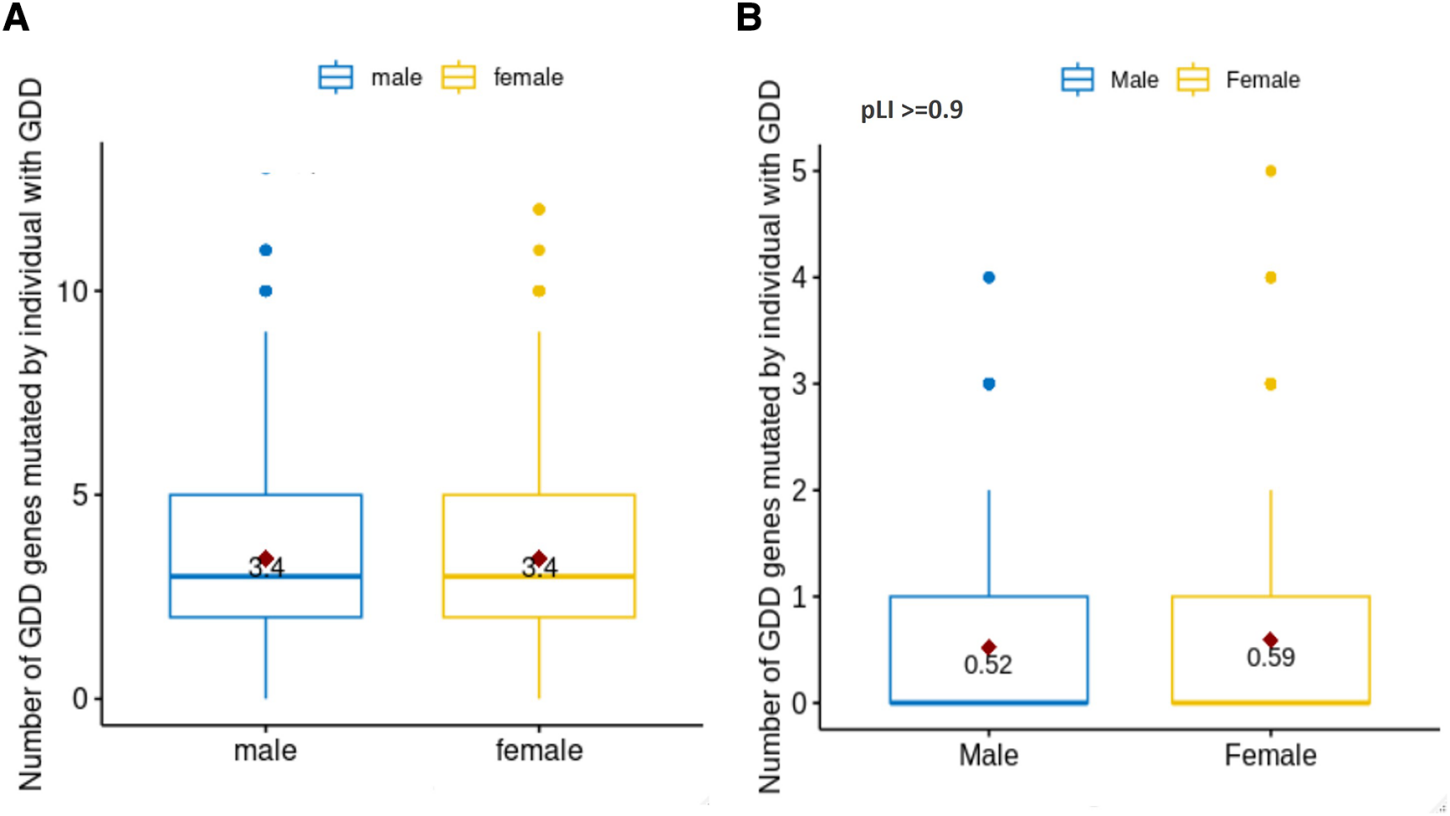
Number of pathogenic mutations in males vs. females with GDD. We identified the number of pathogenic mutations per individual with GDD based on sex (male = blue, female = yellow). (**A**) For all GDD genes. There was no statistical difference between males and females (*T*-test *p* = 0.84). (**B**) For pLi score >= 0.9. The difference between males and females was statistically significant (T test *p* = 1.7E-04).

We wanted to assess if differences in mutation load were present in GDD as previously reported in ASD (50). We therefore quantified the number of pathogenic mutations per individual in each sex (Figure 3A). We observed a similar number of pathogenic variants in GDD/ID genes per individual in males and females (median = 3, *P* = 0.84). We also considered GDD/ID genes present in both sexes but biased toward one sex (Supplementary Table S4). None of the genes that are significantly different between the two sexes are X-linked. Finally, we analyzed the genes having a higher probability (>0.9) of loss-of-function (LoF) intolerance (pLI) score. High pLI genes have been shown to have a higher impact on intellectual quotient in copy number variants in individuals with developmental differences (51, 52). Interestingly, we found that for those with high pLI genes, females had a significantly higher number of pathogenic variants per individual (0.59 vs. 0.52 for male, *p* = 1.7E-4) (Figure 3B).

**Figure 4:**
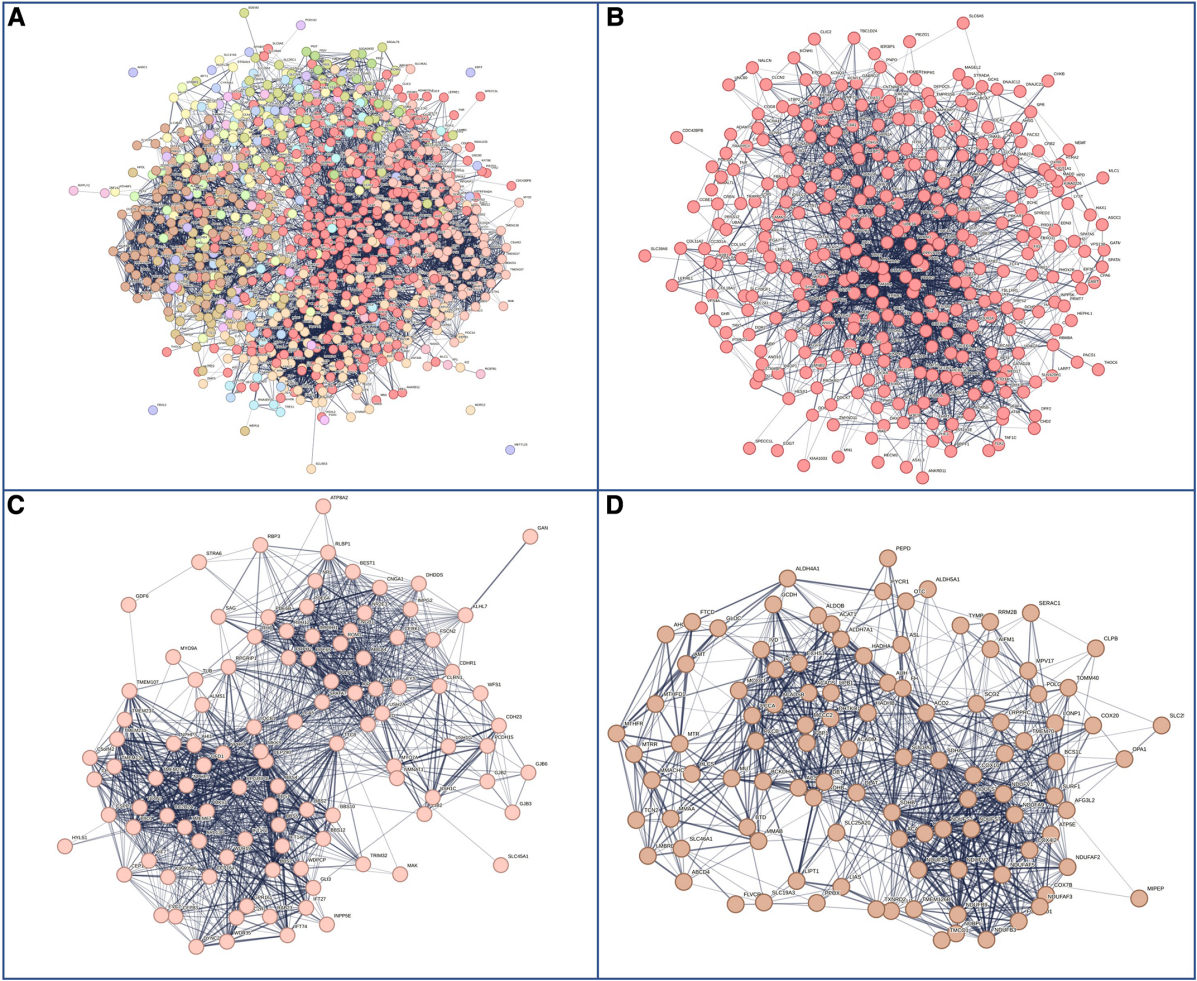
Genes with mutations present in both males and females. (**A**) Overall representations of the genes found to have pathogenic mutations in both males and females with GDD. Each color represents a gene subcluster. (**B–D**) Representative sub-network of more closely related genes. Colors are specific for each sub-cluster.

**Figure 5:**
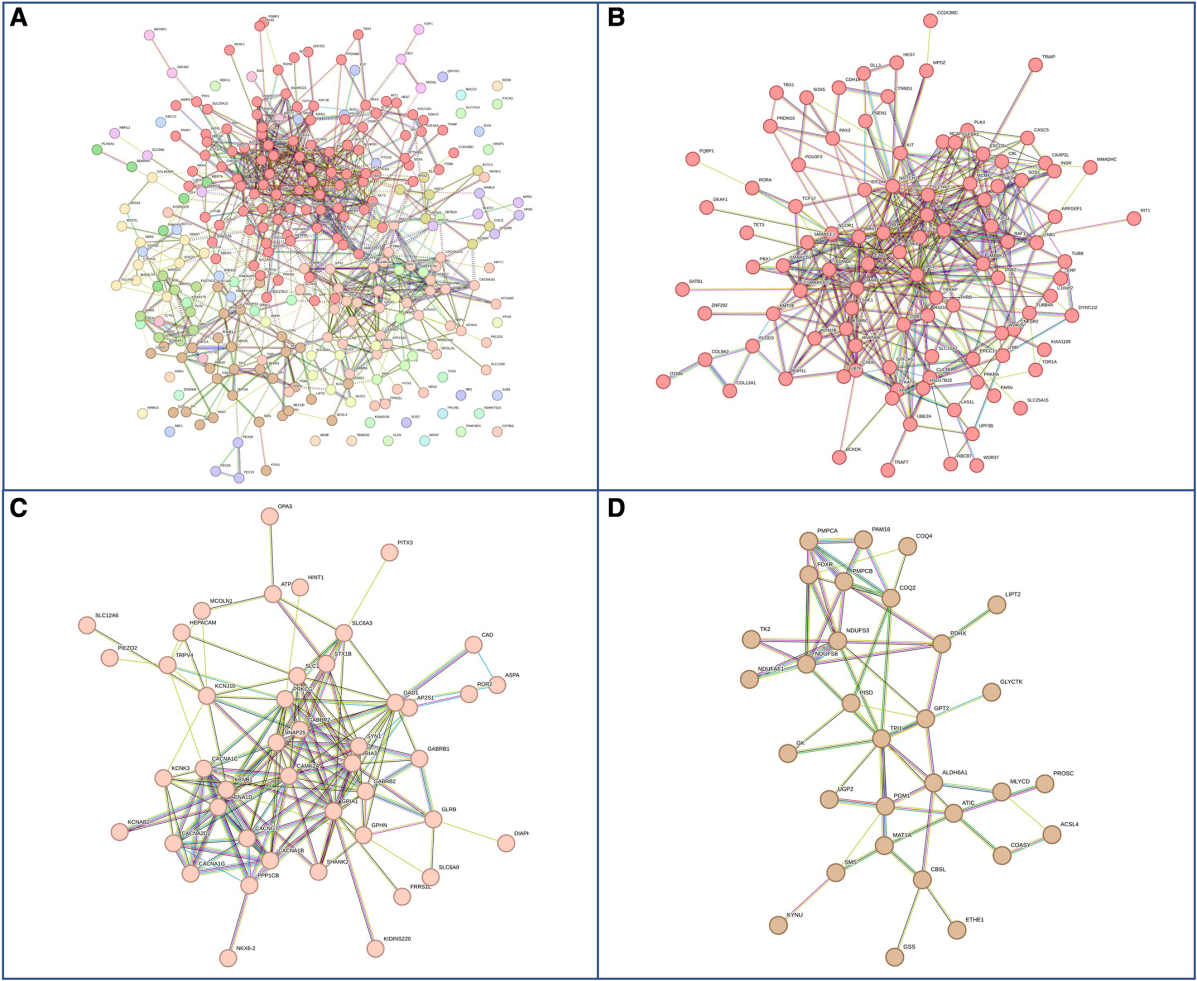
Genes with pathogenic mutations found specifically in males with GDD. (**A**) Overall network representations of GDD genes identified uniquely in males. Each color represents a gene subcluster (**B–D**) Representative subnetworks of more closely related genes. Colors are specific for each sub-cluster.The color of the cluster corresponds to that of figure A.

**Figure 6:**
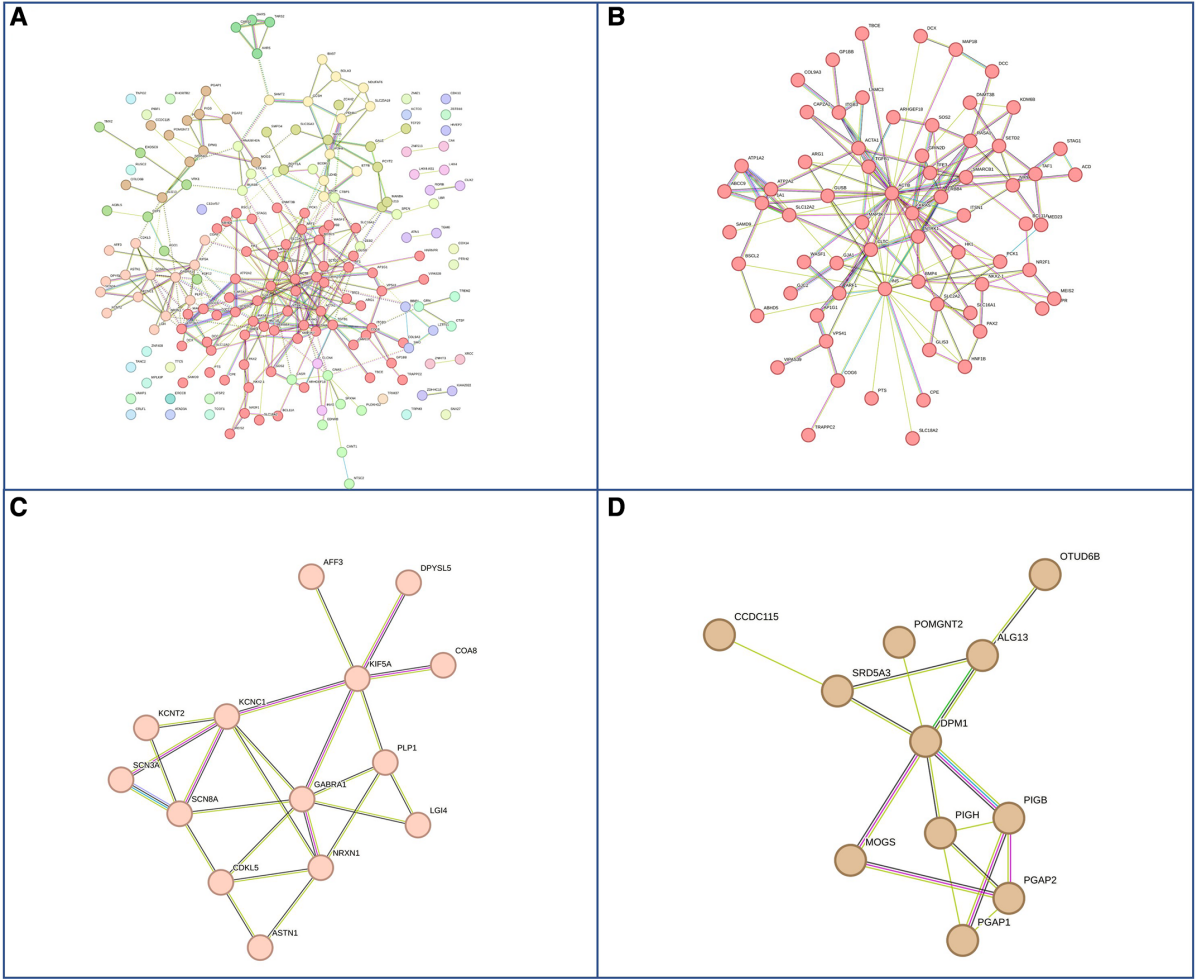
Genes with pathogenic mutations found specifically in females with GDD. (**A**) Overall network representations of GDD genes identified uniquely in females. Each color represents a gene subcluster (**B–D**) Representative subnetworks of more closely related genes. The color of the cluster corresponds to that of figure A.

Next, we hypothesized that differences in phenotypes could be related to differences in gene networks and molecular pathways. GDD genes carrying mutations present in both males and females subdivided in 27 sub-networks and 12 with more than 10 genes (Figure 4A). The first 3 sub-clusters, with the largest number of genes are shown in Figures 4B–D, which were involved in protein binding, chromatin binding and transcription factor; RNA polymerase, DNA binding; and catalytic activity in mitochondria (Supplementary Table S6). On the other hand, GDD genes found mutated specifically in males (Figure 5) also subdivided in 20 sub-networks including 4 with more than 10 genes. In the same way the first 3 sub-clusters were involved in DNA binding; gated channel activity; catalytic activity in mitochondria (Supplementary Table S7). Genes found mutated specifically in females (Figure 6), subdivided in 18 groups and the first 5 with more than 10 genes were associated with protein binding; gated channel activity; Endoplasmic reticulum activity; and mitochondrial enzymatic activity. So there were no clear cut differences in type of molecular function or cellular component between genes specific to males vs. females. (Supplementary Table S8).

### 3.3. Phenotypic differences and gene expression profiles in males and females with GDD

We hypothesized that phenotypic differences in individuals with GDD may be related to differences in sex-biased gene expression in the brain, as was postulated before for ASD (53, 54). We leveraged sex-specificed gene expression data from two datasets: GTEx (42) and BrainSpan (43).

**Figure 7:**
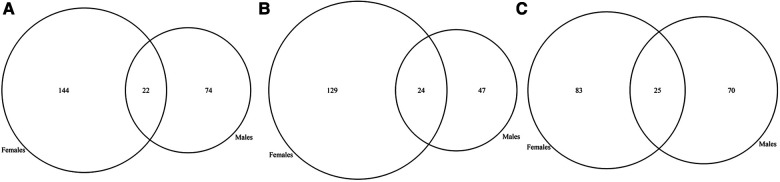
Venn diagrams for significant enriched GO pathways in (**A**) amygdala, (**B**) cerebellar hemisphere, and (**C**) hippocampus.

Firstly, we wanted to assess if regional differences in gene expression could explain phenotypic differences in GDD as seen recently in ASD (55). We assessed the gene expression profile by sex in each brain region, while adjusting for the GDD genes found mutated in males vs. females specific (and enriched) in the individuals with GDD (Supplementary Figure S1). We took advantage of the GTEx data in 11 brain regions (Amygdala, Caudate, Cerebellar Hemisphere, Cerebellum, Cortex, Hippocampus, Hypothalamus, Nucleus accumbens, Putamen, Spinal cord, and Substantia nigra) and 2 cell lines (Frontal Cortex (BA9) and Anterior cingulate cortex (BA24)). Using a linear mixed model of the logarithm of gene expression (See Methods), we found significant associations for the sex in all the considered brain regions. Moreover, when assessing the effect direction we observed that only in the amygdala, the anterior cingulate cortex, and the nucleus accumbens, being a male significantly decreases the gene expression, while for the remaining brain regions the association is positive. These results were globally confirmed when considering only sex specific genes (Results not shown). The results for all genes were reported in Supplementary Table S8. Then, we constructed gene expression clusters for sex specific genes and found different biological processes involved between the two sexes (See Methods). For example, focusing on the amygdala, the cerebellar hemisphere and the hippocampus, brain regions known to be strongly associated in emotions, higher cognitive functions, learning and memory, respectively, we found a weak overlap in GO pathways enrichments between males and females (Figure 7; Supplementary Figures S2-S7). Collectively, we found that sex is associated with gene expression across a large set of brain regions pointing to different biological processes between males and females. However, the crossed effect of sex and developmental stage on gene expression remains to be understood.

Another way genes can influence phenotypic presentation is via timing of expression in brain development as seen before in ASD (56). In order to address the role of the crossed effect of sex and the developmental stage on gene expression, we leveraged information from the BrainSpan database (43) for 19 brain regions [amygdaloid complex, anterior (rostral) cingulate (medial prefrontal) cortex, cerebellar cortex, dorsolateral prefrontal cortex, hippocampus (hippocampal formation), inferolateral temporal cortex (area TEv, area 20), mediodorsal nucleus of thalamus, orbital frontal cortex, posterior (caudal) superior temporal cortex (area 22c), posteroventral (inferior) parietal cortex, primary auditory cortex (core), primary motor cortex (area M1, area 4), primary somatosensory cortex (area S1, areas 3,1,2), primary visual cortex (striate cortex, area V1/17), striatum, ventrolateral prefrontal cortex, cerebellum, dorsal thalamus, and primary motor-sensory cortex (samples)]. We integrated the developmental stage in our model, while adding an interaction term between the developmental stage and sex to highlight synergies between these two variables on gene expression patterns. It is worth noting that we removed the dorsal thalamus, cerebellum, and primary motor-sensory cortex (samples), since we did not have complete data for our analysis. Firstly, we found that developmental stage is positively associated with gene expression in 4 brain regions: the amygdaloid complex, the cerebellar cortex, the striatum, and the hippocampus. These 4 regions have been shown to be strongly involved in a large range of developmental disorders (57–61). Moreover, sex-developmental stage interaction is significantly positively associated in the dorsolateral prefrontal cortex and the posterior (caudal) superior temporal cortex (area 22c) brain regions. Results were recapitulated within Supplementary Table S10. Consistent with results found in the GTEx data, these findings suggest that developmental timing may vary across sexes, resulting in different gene expression patterns among males and females, leading to phenotypic variations between sexes.

### 3.4. GDD associated with autism and sex differences

**Figure 8:**
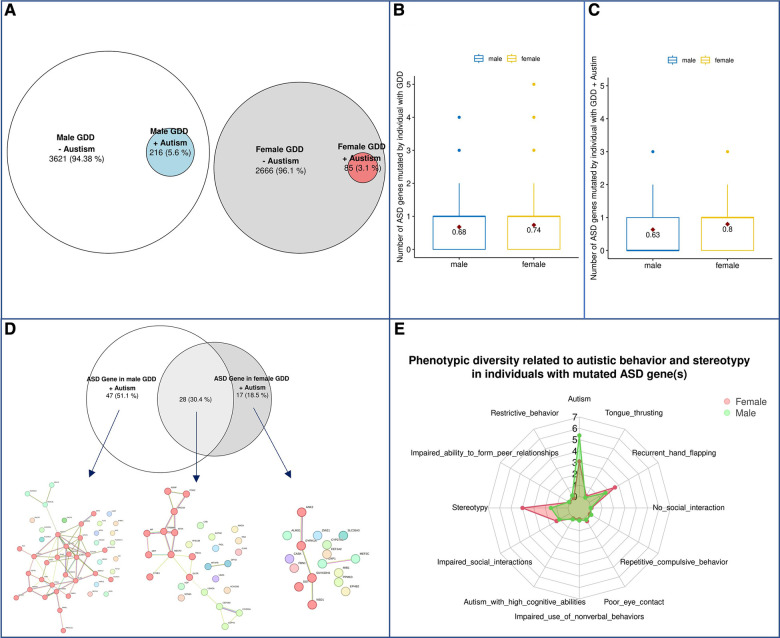
Phenotypic differences in autism spectrum disorder. (**A**) Schematic representation of the population. (**B**) Prevalence of ASD genes in males and females with GDD overall. (**C**) Prevalence of ASD genes in males and females with GDD + Autism. (**D**) ASD genes in GDD + Autism. ASD genes (92) were found in 216 ♂ Males and 85 ♀females. 47 (51.1%) were unique to males and 17 were unique to females with 28 genes found in both sexes. A global representation of ASD gene networks were identified for each with a specific color for each subcluster highlighted by MCL clustering. Each color represents a gene subcluster (**E**) Phenotypic diversity related to autistic behavior and stereotypy in GDD individuals with mutated ASD gene(s).

Next, we assessed a subgroup of individuals with GDD also presenting with autism, where sex differences have been observed (62). Autism is significantly overrepresented in males with GDD (5.62% in males vs. 3.09% in females *P* = 4.22E-04) (Figure 8A), but not in the 3–4:1 (62) found in the previous reports investigating individuals with ASD. First, we assessed if autism was more prevalent in males with GDD due to increased ASD gene mutations in males. We found that, actually, females had a higher mutation rate in ASD genes than males (0,74 in females vs. 0,68 in males; *p* = 0.0043, *T*-test) (Figures 8B,C). We then assessed if different types of ASD genes were mutated in males vs. females with GDD and autism. We found that 28 ASD genes were mutated in both sexes while 47 and 17 were unique to males and females, respectively (Figure 8D). We then examined their gene network (Figure 8D). The ASD genes shared by males and females were subdivided into 3 subnetworks with only one with more than 10 genes. Subcluster1 is related to chromatin binding, and it may be noted that one of the biological processes is memory (Supplementary Table S11). The ASD genes that are specifically mutated in males are divided into two subnetworks, one of which is larger and enriched for genes involved in voltage-gated ion channel activity and regulation of cell signaling, transport and activity (Supplementary Table S11). We can also note processes related to the synapse, neuron and dendrite. On the other hand, female specific genes did not show as much interaction between genes with only 2 small sub-networks with low functional enrichment which is related to methylation (Supplementary Table S11).

Next, we wondered whether sex could influence phenotypic presentation for the same gene. We tested if the phenotypic presentation of individuals with GDD was found to have pathogenic variants in ASD candidate genes (Supplementary Table S2) differed by sex. Interestingly, we found that males presented with autism, whereas females presented with an increase in stereotypies (Figure 8E). Similar results to those described above were obtained for genes with a high pLI (Supplementary Figure S8).

## 4. Discussion

Sex differences are increasingly recognized in several disorders. A better understanding of sex difference has the potential to help personalized interventions, but also shed light on the molecular underpinning of disorders, assist in precision medicine and ultimately refine targeting of participants in clinical trials. While differences in both prevalence and clinical manifestations have been recognized in ASD (3, 63–65), there are no reports, to our knowledge, of phenotypic differences in GDD, which affects 3% of the pediatric population, with the exception of X-linked conditions such as Fragile X syndrome (FXS). Fortunately, a large cohort of individuals with GDD, the Deciphering Developmental Disorders (DDD), has been developed and can help delineate how sex can influence phenotypes in GDD. The DDD consortium included individuals with neurodevelopmental differences such as ID, GDD and ASD with no known etiology from the age of birth to 82 years old.

We analyzed the DDD cohort and found only a small predominance of males (3,838 males and 2,750 females) with GDD diagnosis. We would hypothesize that the equal prevalence in DDD may be related to excluding individuals with male prevalent known causes (Fragile X syndrome for instance, which is the most common single gene cause in males). The representation of phenotypes can also affect the prevalence, with language delay being especially more impacted in males (66). Nonetheless, we show for the first time significant sex differences in the type of phenotypes between males and females including autism, stereotypies, macrocephaly or microcephaly as well as distinctive facial features such as downslanted or upslanted palpebral fissures. More analysis will be needed to explain the different morphological traits found depending on the sex. We focused on functional phenotypes as they could be modified with interventions in the future. We should also be reminded that there are limitations when using the DDD database for analyzing dysmorphic features. Annotating each individual's dysmorphic features exhaustively and accurately is challenging. While extensive phenotypic information is provided for some DDD participants, it is not the case for all participants. Certain dysmorphic features that show significant differences may be more representative of specific syndromes that are more prevalent in one sex rather than being inherent features themselves.

The vast majority of GDD/ID genes could affect either males or females. Surprisingly, we found that while genes specifically mutated in males vs. females with GDD differed significantly, they presented with overlapping molecular functions. This makes sense considering that we focused the study on a shared condition, GDD, which would imply a set of molecular pathways. Although they used different analysis methods, two genes (DDX3X, EEF1A2) from our list, with a higher prevalence observed in females, have been also observed by Turner et al. 2019 (67).

Females with ASD have been shown to present with a higher mutation load than males with ASD, but we did not observe that for GDD genes in individuals with GDD. But, we show for the first time that females with GDD presented with an increased load when considering genes with lower tolerance to mutation (reflected by high probability of intolerance to loss-of-function (pLI) specifically. This may suggest that women are equally affected by GDD genes in general but especially resistant to high pLI genes, which could be assessed in ASD in other cohorts.

Gene expression patterns of GDD genes could also contribute to sex differences in GDD. Indeed, gene expression profiles for GDD genes were significantly different by sex, consistent with the idea that for a gene to lead to a condition, it must correspond to the gene expression profile relevant to the sex of the individual (68) We found that gene expression for the GDD genes specifically mutated in males and females was significantly different in two brain regions, cerebellar cortex and mediodorsal nucleus of thalamus, respectively. Interestingly, these two regions have been shown to be associated with autism and cognitive disabilities (55, 69). This result is of particular interest since autism is one of the phenotypes we found significantly different in our study. Moreover, in the cerebellar cortex we observed significant pathway enrichments in male specific clusters for channel-related processes, while female specific clusters show synapse-associated pathway enrichments, pathways known to be involved in autism spectrum disorders (70, 71). This result suggests that phenotypic differences operating between sexes may be explained by differences in pathways of genes expressed in specific regions of the brain (which are sex-biased). We also found that sex influenced developmental aspects of gene expression for GDD genes mutated in males and females in the dorsolateral prefrontal cortex and the posterior (caudal) superior temporal cortex (area 22c) brain regions which could also explain differences in clinical manifestations.

There is mounting evidence that phenotypic complexity may be associated with genotypic complexity in ASD (72). So we assessed if genotypic differences could be found in individuals with GDD and associated autism phenotype.We found that while the prevalence of ASD was higher in males than females with GDD, the mutation load was significantly higher in females overall, as reported before as an explanation for the female “protective” effect (3, 4, 65, 73). But we also observed that females with ASD genes presented differently than males with more stereotypies and less autistic traits, suggesting that when considering additive effect (74) (more mutations leads to different phenotype), sex should be considered also as a modifier. This is different from previous findings in ASD cohorts which found that repetitive behavior and limited interest were more common in boys after the age of 5 (75). This difference may be related to the fact that the individuals in our cohort harbored GDD. Other sex differences in ASD previously reported included increase in sensory input reactivity in females but this was not reported in the DDD cohort (76).

Furthermore, future work will be needed to understand why stereotypies were increased in females with GDD presenting with ASD genes, contrary to the previous reports of increased internalizing behavior and decreased stereotypic/repetitive behaviors compared to males (77).

More research will need to test these findings in other populations to assess the impact of genetic background on sex-difference in phenotypes. In addition, it will be important to probe if treatments aimed at genes shared between males and females return a similar response. Our study will also inform pre-clinical studies, in which it may be important to consider sex-specific (or at least sex diverse) models. Our study is also based on a list of candidate genes for GDD and ASD. The genes had to be identified in 3 independent publications to be included in the candidate gene list. This represents an association and that we can not ascertain if there is a causal relation as some may not have supporting basic science evidence. It is important to note that multiple genes are also associated with other phenotypes and therefore present with complex phenotypes which may make causation more challenging to assess. We will need to be expanded to more genes as they become available. Moreover, other genomic events, CNV, chromosome abnormalities and genes not specifically involved in NDD may also influence phenotypes in a sex-specific manner and should be considered in the future. It is also important to note that the use of HPO has the advantage, as a research tool, of standardizing the ontology and allowing sharing between disorders and models, but also some trade-offs in terms of precision in describing fine clinical entities. For example in our case we found autism and autistic behavior in GDD individuals but these terms can be confusing in a clinical context. Autism in HPO is defined as: “The term refers to the diagnosis of autism and is left for convenience. However, it is preferable to annotate the exact phenotypic abnormalities rather than merely the diagnostic category autism.”

The participants recruited in DDD based on not having already a genetic diagnosis, limiting our results to individuals with GDD of unknown etiology. Although we obtained some insights into gender differences among individuals with GDD, more research on other cohorts of individuals with GDD will be needed. Those could be expanded to included individuals with known causes. Special attention to distinctive features (dysmorphic features) should be considered also and mechanisms put in place to established comprehensive phenotypic labeling.

Sex seems to be an important variable to consider when assessing the molecular basis of GDD and will benefit from further research. While additive genetic mutation may explain neurodiversity in GDD in part, sex specific differences in patterns of gene expression should also be considered. Finally, modeling of GDD genes should take into account the sex in whom patients presented with those genes.

## Data Availability

Publicly available datasets were analyzed in this study. This data can be found here: https://www.ddduk.org/access.html, https://ega-archive.org/studies/EGAS00001000775: EGAD00001004390 EGAD00001004388.
