# Supplementary material for: Sex difference contributes to phenotypic diversity in individuals with neurodevelopmental disorders
Source: Front Pediatr. 2023 Aug 7;11:1172154. doi: 10.3389/fped.2023.1172154 (PMC10441218; doi:10.3389/fped.2023.1172154)
Supplement: Supplementary file 12 [file Datasheet1.docx]

*Supplementary Material*

**SUPPLEMENTARY METHODS**

### **Genomic sequence analysis**

The exome data pertaining to GDD individuals from DDD were aligned to the GRCh37/hg19 built reference genome. We reprocessed the genomic sequences and re-aligned them on the GRCh38/hg38 reference genome. Short genomic variants which include single nucleotide variants (SNVs) and indels were called using germline variant calling pipeline by implementing the best practices from GATK. Figure S1 presents the overall workflow which comprises two steps: (a) variant calling and (b) annotation and filtering.


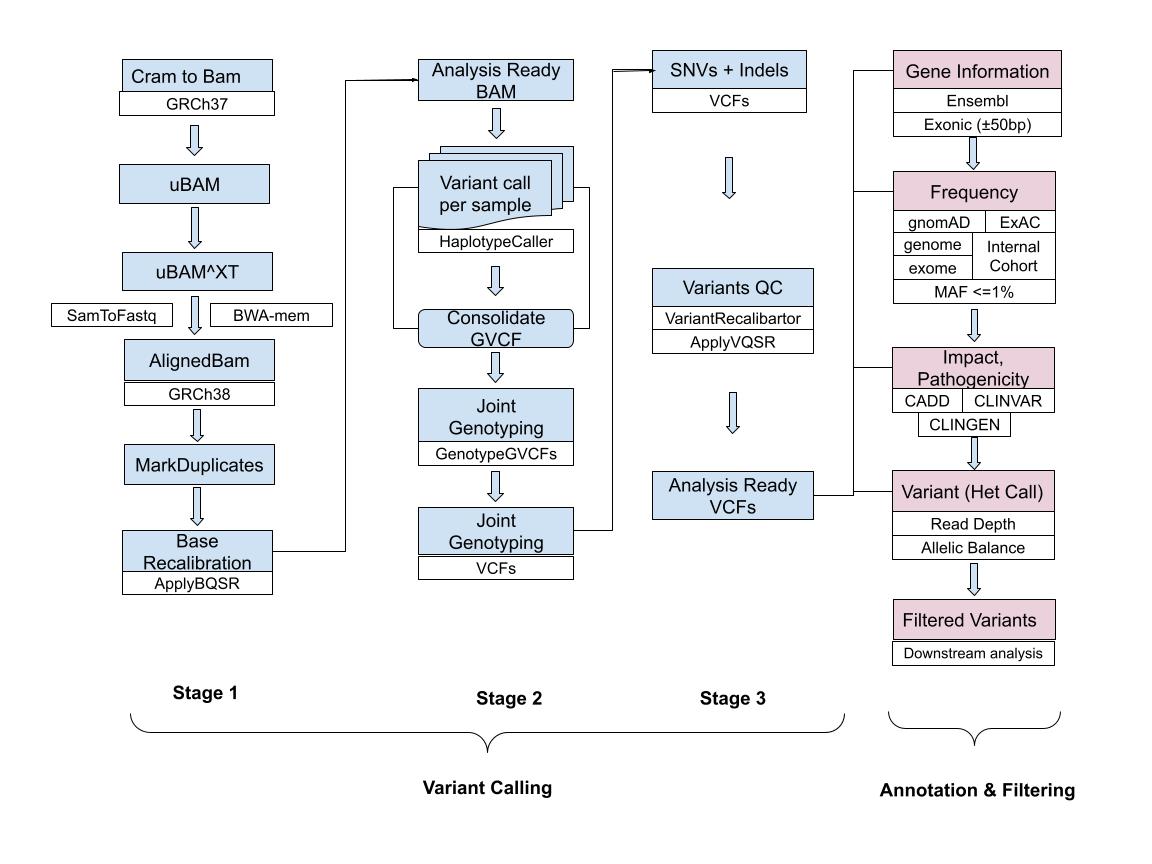


**Workflow for joint variant calling, annotation and filtering.** The first part (colored in blue) of the workflow represents the variant calling. It has three substages where in stage 1 the GRCh37/hg19 aligned BAM files are aligned to the GRCh38/hg19 reference genome. In the second stage, the variants are called per sample and then jointly genotyped for all the samples in the cohort. Finally, in the third stage, the joint genotyped variants in the callset are subjected to filtering and recalibration based on quality. In the second part (coloured in pink), the annotation and filtering strategies are applied. The quality enriched variants for the callset are annotated for information related to gene position, population level frequency, impact and pathogenicity.

The variant calling step has further three stages: (1) alignment of GRCh37/hg19 samples to GRCh38/hg38 reference built and aligned reads are de-duplicated and recalibrated using GATK based tools. (2) The GRCh38/hg38 aligned samples are subject for variant calling using GATK-HaplotypeCaller for the exonic regions (±250 bp) present in the target enrichment file. Post variant calling step, for all these samples (for the given cohort) were jointly genotyped using GATK-GvcfGenotyper in cohort mode. Finally, in (3) the jointly genotyped variant callset were subjected to filtering, annotation and variant quality recalibration using GATK based tools VariantRecalibrator and ApplyVQSR.

The second step of the workflow comprises annotation and filtering of all the variants across all the samples present in the cohort. The variants were annotated for gene information (obtained from Ensembl release 100), frequency present in population databases such as gnomAD (genome-v3.0,v2.1.1, exome - v2.1.1), ExAC (liftover GRCh37/hg19) and GDD internal cohort and pathogenicity scores from databases such as CADD (v1.6), Clinvar/Clingen (release 26/10/2021) and dbNSFP (v4.2.a, downloaded 06/04/2021) which comprises prediction scores from LRT, FATHMM, PROVEAN and MutationTaster, REVEL (v1.3, downloaded on 04/011/2021). The annotation of the variants in the callset were annotated using the Ensembl-VEP program incorporating plugins pertaining to all the above annotation sources.

Finally, the annotated variants are filtered for following criteria:

1. Filtering out variants that fall outside the exonic regions (±50bp).
2. MAF <=1% in gnomAD genome (v3) OR gnomADg (v2.1.1) OR gnomADe(v.2.1.1) OR ExAC
3. Keeping variants with transcript having impact:

MODERATE or HIGH OR splice_donor_5th_base_variant OR splice_donor_0th_base_variant OR splice_donor_region_variant OR splice_polyprimidine_tract_variant OR CLNSIG

1. Transcript prioritization based on variants falling in protein_coding region, having HIGH OR MODERATE effect impact, canonical rank and transcript length.
2. Variants were filtered for pathogenic/likely_pathogenic in CLINVAR/CLINSEG.
3. Pertaining to quality, the variants per individual were filtered for
   1. Read Depth >=5
   2. Heterozygous calls having allelic balance [0.15,]

### **Candidate gene list**

The last update of the gene list is 9 December 2022 for all the databases (SysID, DisGenet, HPO, OMIM, Orphanet, Phenolyzer, Open Targets, AutDB)

SysID : latest update on November 18th 2021

DisGenet : version 7.0, January 2020

HPO: 2022-10-05 Release

OMIM: 2022-12-09 Release

Orphanet: 2022-11-28 Release

Phenolyzer: 2019-08-05 update knowledgebase

Open Targets: release of the Platform - 22.11, 24 November 2022

AutDB : Updated Sep, 2022

**SUPPLEMENTARY FIGURES**


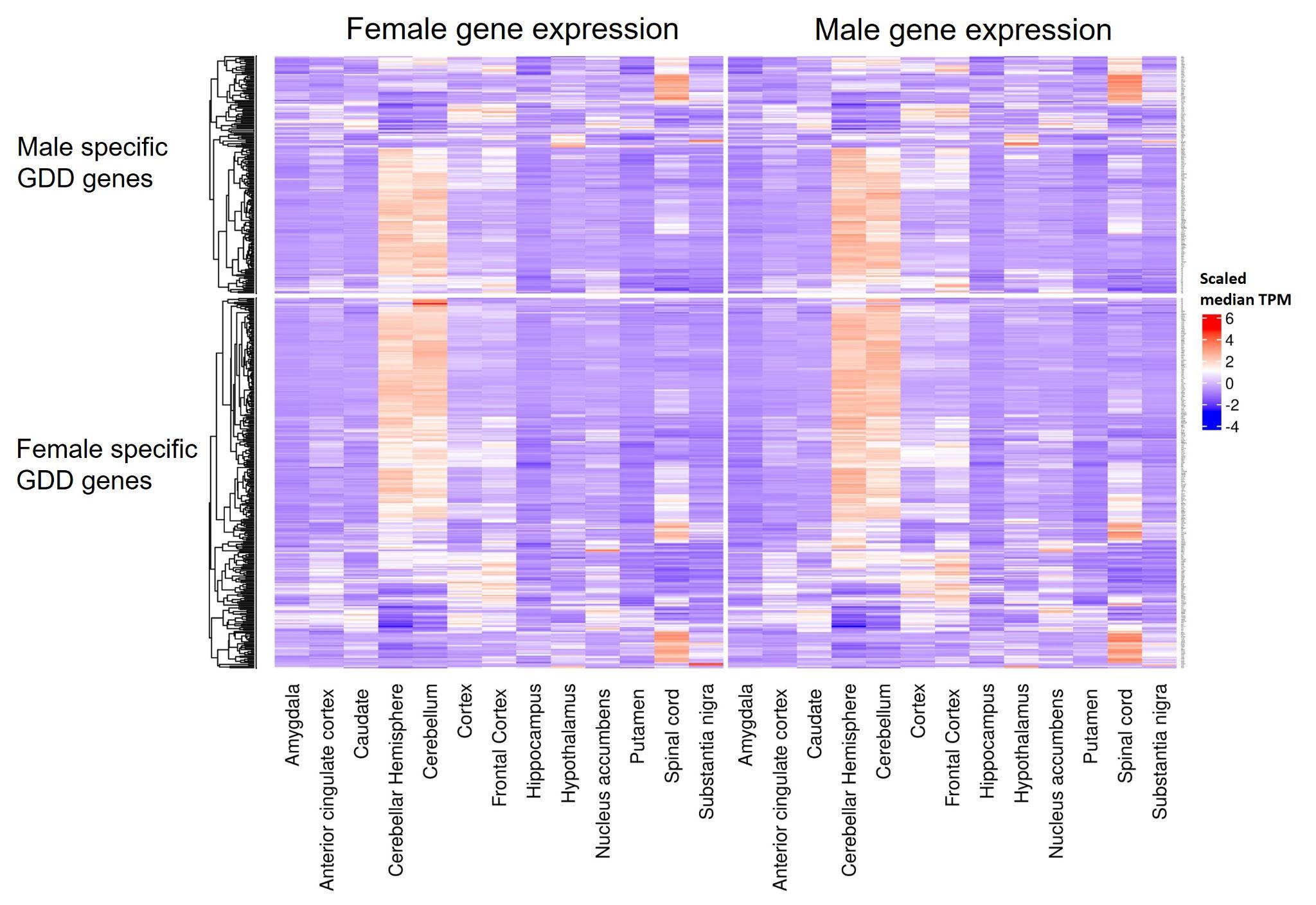


**Supplementary Figure 1. Brain expression of GTEx data in males and females.** Gene expression profile (brain regions) for GDD genes found specifically in males and females. Median tpm's are scaled by gene with z score transformation.


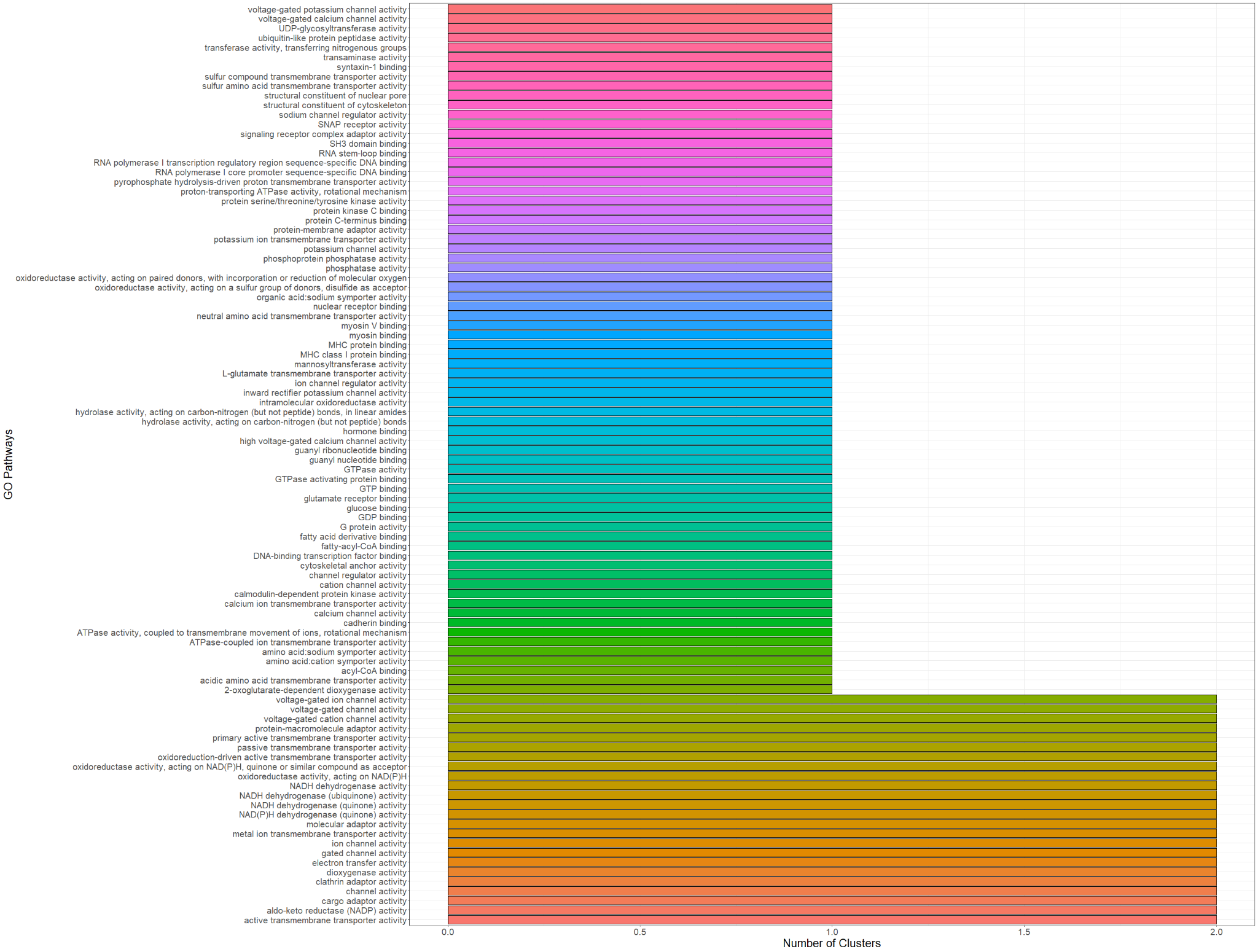


**Supplementary Figure 2. Gene-Ontology enrichment for male-specific gene clusters in the amygdala. Here we focused only on significant biological pathways**.


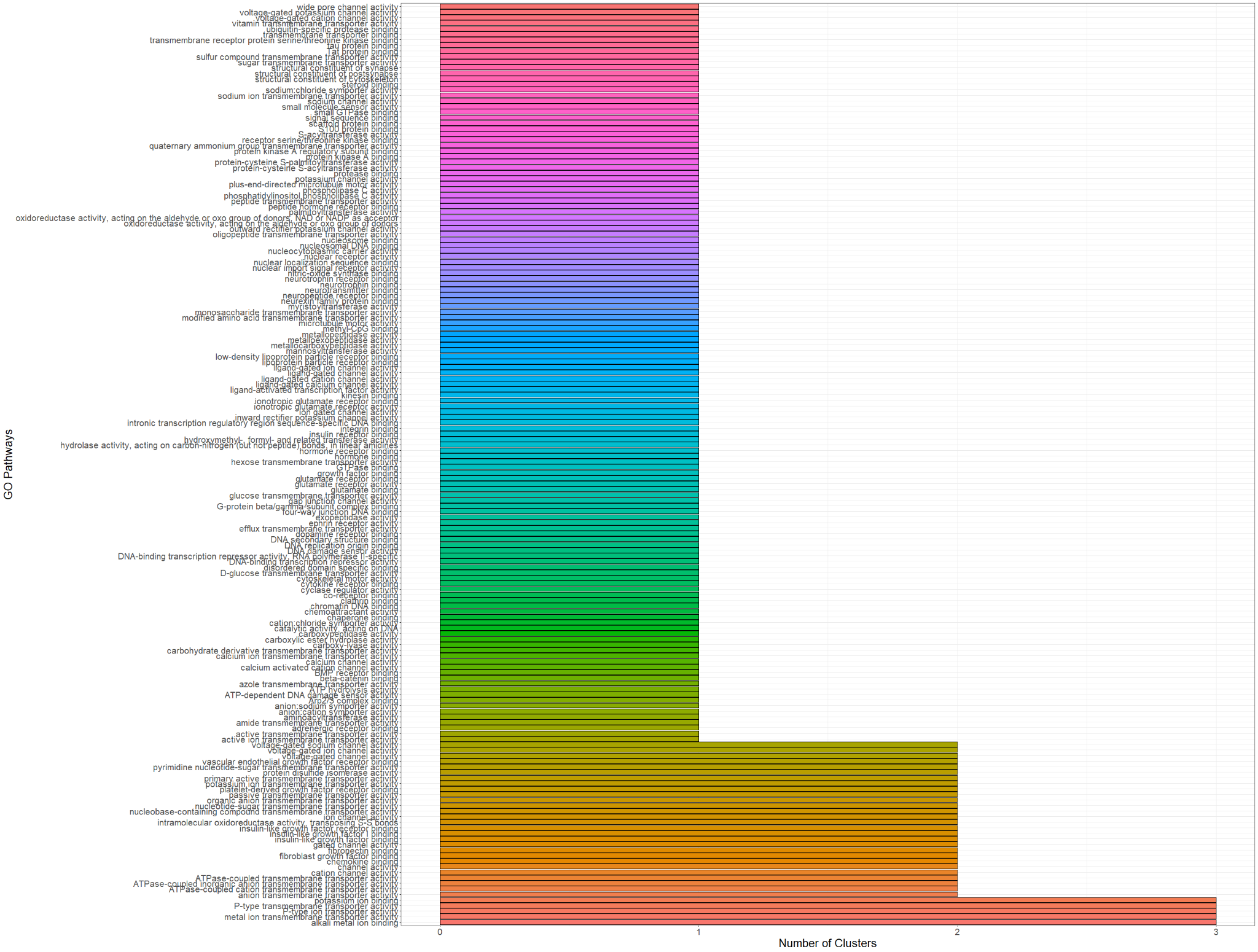


**Supplementary Figure 3. Gene-Ontology enrichment for female-specific gene clusters in the amygdala. Here we focused only on significant biological pathways**.


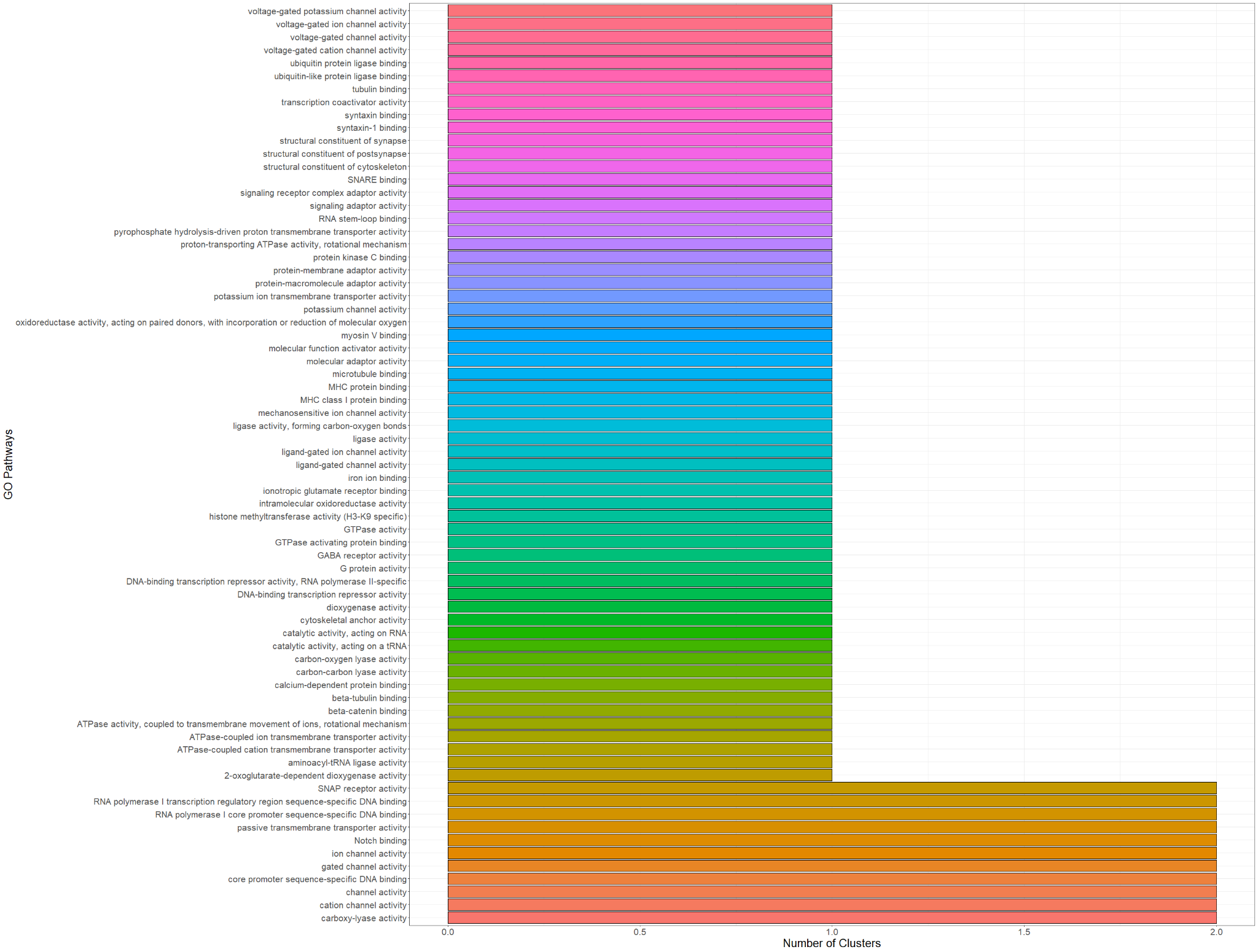


**Supplementary Figure 4. Gene-Ontology enrichment for male-specific gene clusters in the cerebellar hemisphere. Here we focused only on significant biological pathways**.


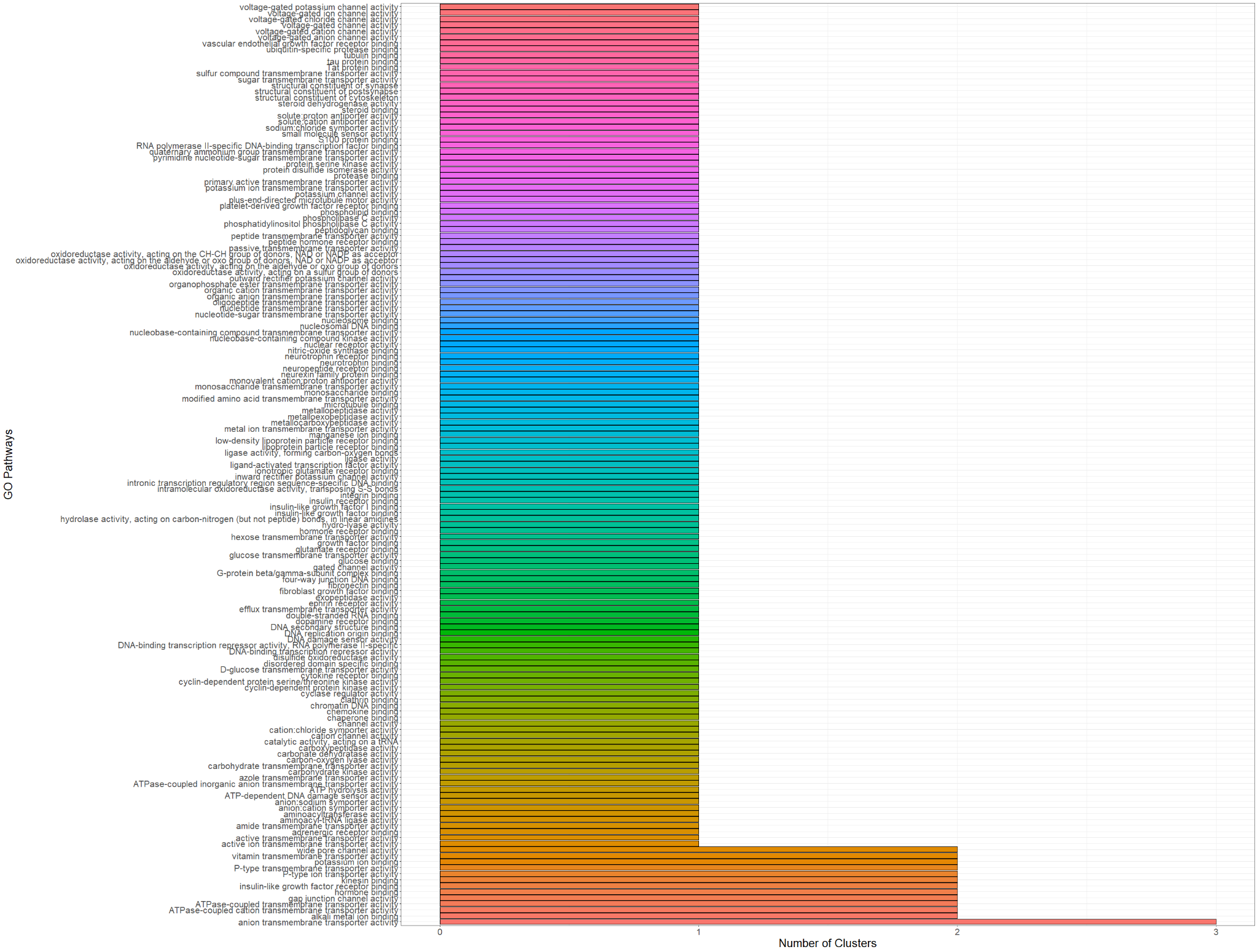


**Supplementary Figure 5. Gene-Ontology enrichment for female-specific gene clusters in the cerebellar hemisphere. Here we focused only on significant biological pathways**.


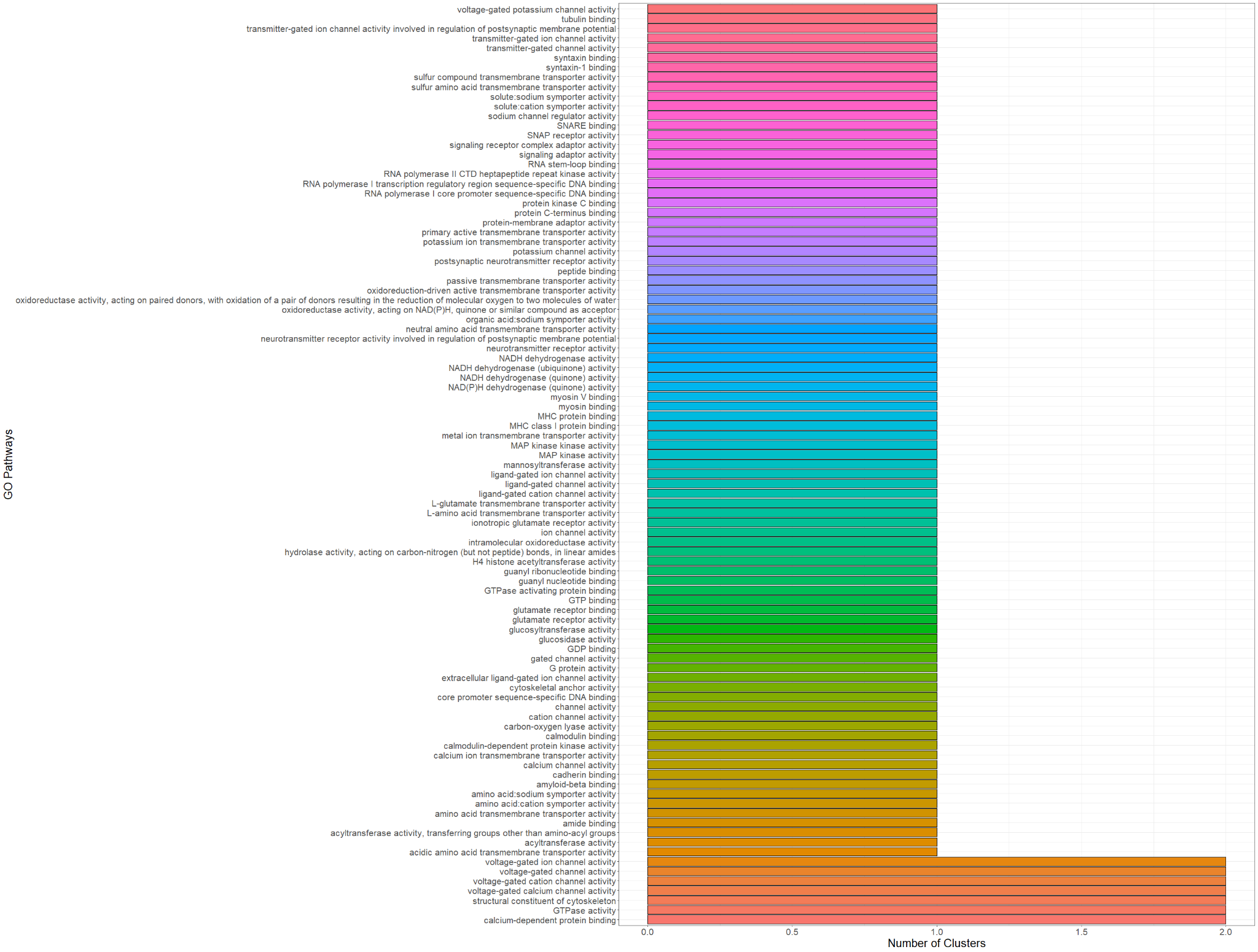


**Supplementary Figure 6. Gene-Ontology enrichment for male-specific gene clusters in the hippocampus. Here we focused only on significant biological pathways**.


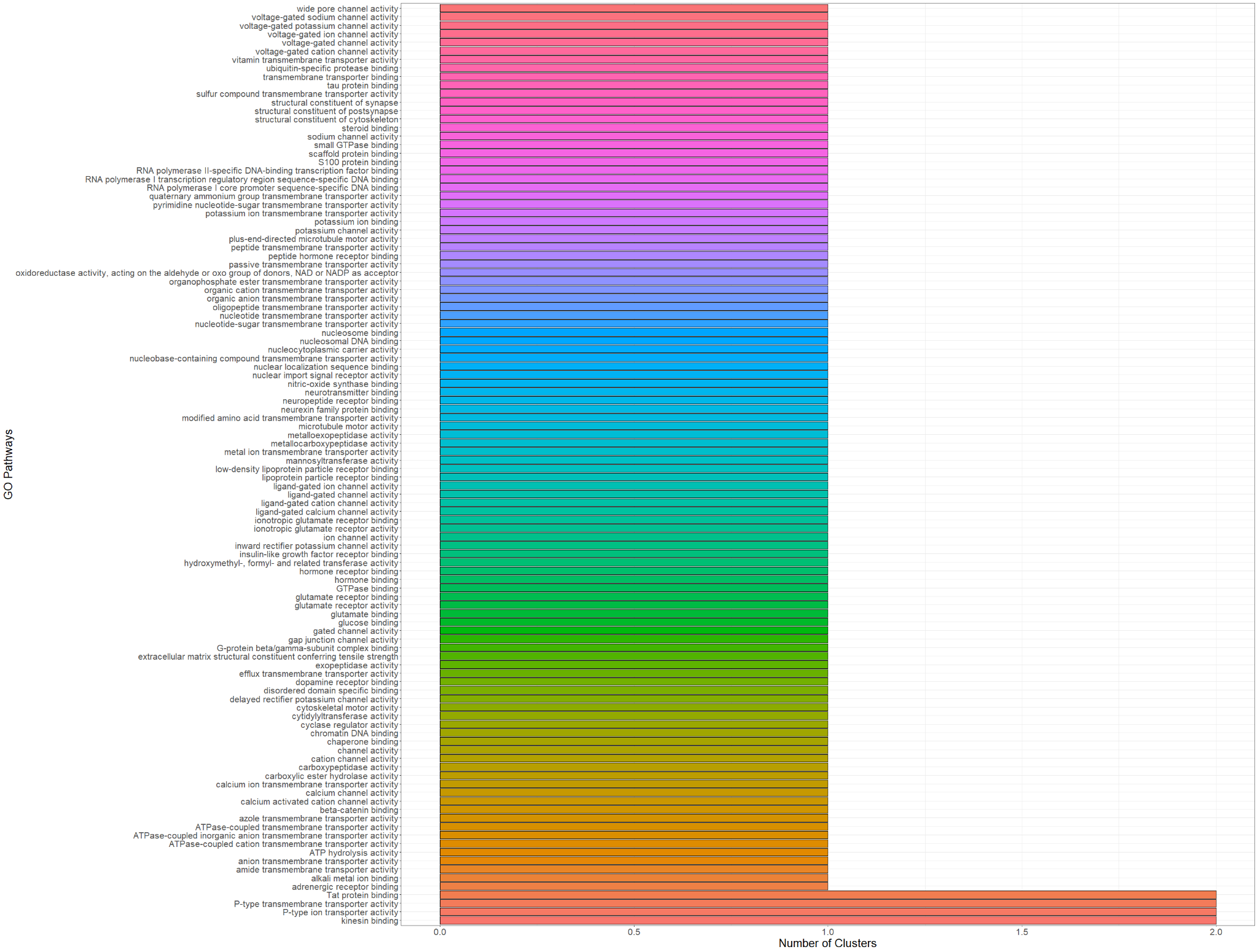


**Supplementary Figure 7. Gene-Ontology enrichment for female-specific gene clusters in the hippocampus. Here we focused only on significant biological pathways**.


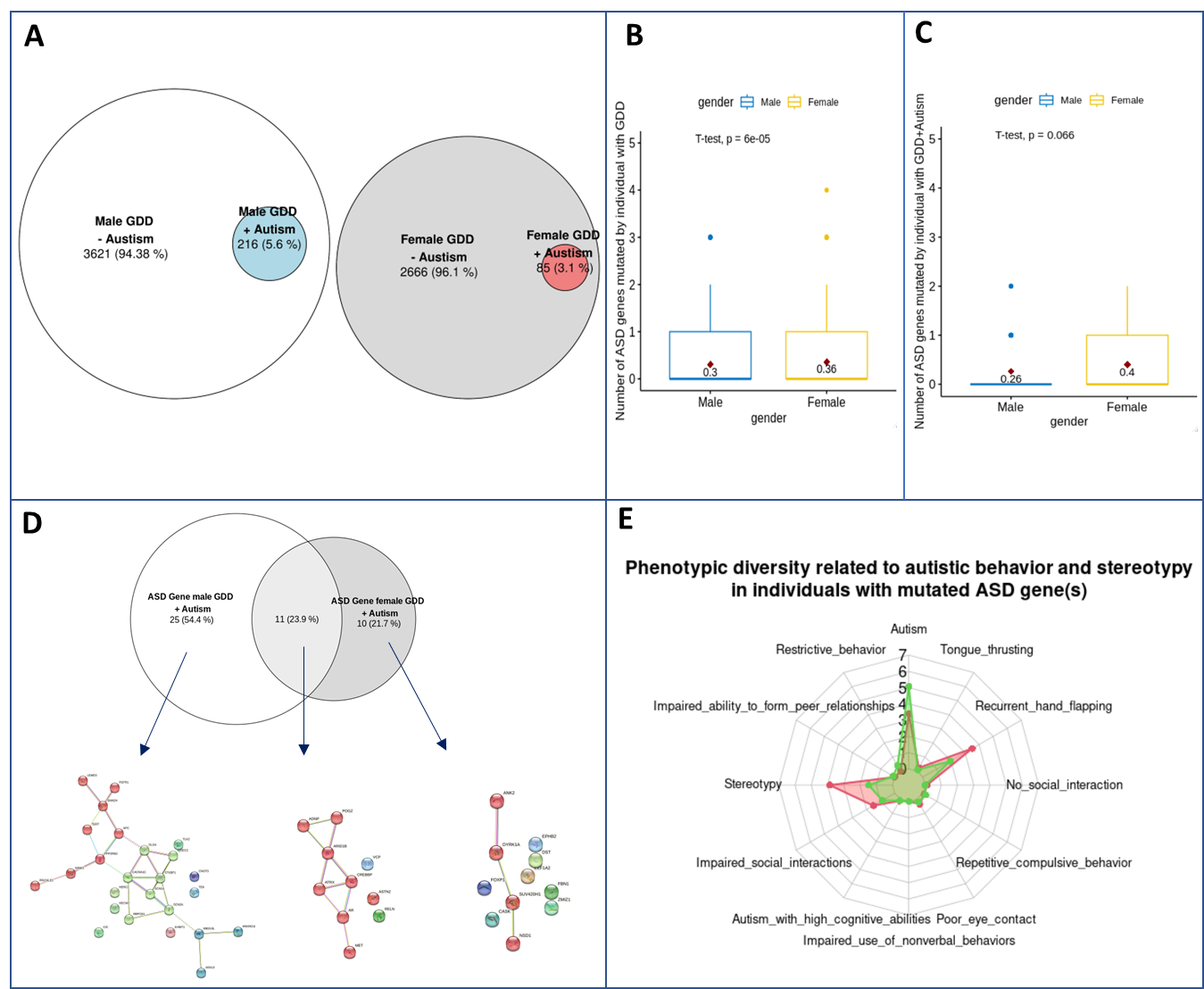


**Supplementary Figure 8. Phenotypic differences in autism spectrum disorder for genes with high pLI.** (**A)** Schematic representation of the population. **(B)** Prevalence of ASD genes in males and females with GDD overall. **(C)** Prevalence of ASD genes in males and females with GDD + autism. **(D)** ASD genes in GDD +Autism. ASD genes (92) were found in 216 ♂ Males and 85 ♀females. 25 (51.1%) were unique to males and 10 were unique to females with 11 genes found in both sexes. A global representation of ASD gene networks were identified for each with a specific color for each subcluster highlighted by MCL clustering. **(E)** Phenotypic diversity related to autistic behavior and stereotypy in GDD individuals with mutated ASD gene(s).

**SUPPLEMENTARY TABLES**

**Supplementary Table 1: Candidate gene list for Intellectual Disability and Global developmental delay.** Intellectual Disability and Global developmental delay candidate genes list was developed by identifying genes found in at least 3 independent references.

**Supplementary Table 2. Candidate gene list for autism spectrum disorder.** Autism spectrum disorder candidate genes list was developed by identifying genes found in at least 3 independent references.

**Supplementary Table 3: Sex representation of phenotypes identified in individuals with Global developmental delay. Phenotypes significantly more differently represented in male and in female.**

**Supplementary Table 4: Genes with pathogenic mutations most commonly found in males and females with GDD in the DDD cohort.**

**Supplementary Table 6. Results from linear mixed models for phenotypic differences by sex. A) For all phenotype > 5 individuals. B) For fisher significant phenotypes.**

**Supplementary Table 6. Pathways associated with GDD genes shared by males and females with GDD.**

**Supplementary Table 7. Pathways associated with GDD genes shared by males and females with GDD.**

**Supplementary Table 8. Pathways associated with GDD genes shared by males and females with GDD.**

**Supplementary Table 9. Coefficients, Confidence Intervals and p-values for the linear mixed model of the logarithm of the gene expression across the 13 GTEx brain regions.** Models were adjusted for sex specificity while integrating a gene-level random effect.

**Supplementary Table 10.** **Coefficients, Confidence Intervals and p-values for the linear mixed model of the logarithm of the gene expression across the 16 BrainSpan brain regions.** Models were adjusted for sex specificity while integrating a gene-level random effect.

**Supplementary Table 11. Pathways associated with candidate ASD genes mutated in males and females with GDD and autism.**
